# Attempted suicide in Sri Lanka – An epidemiological study of household and community factors

**DOI:** 10.1016/j.jad.2018.01.028

**Published:** 2018-05

**Authors:** D.W. Knipe, D. Gunnell, M. Pearson, S. Jayamanne, R. Pieris, C. Priyadarshana, M. Weerasinghe, K. Hawton, F. Konradsen, M. Eddleston, C. Metcalfe

**Affiliations:** a Population Health Sciences, Bristol Medical School, University of Bristol, Canynge Hall, 39 Whatley Road, Bristol BS8 2PS, UK; b South Asian Clinical Toxicology Research Collaboration (SACTRC), Faculty of Medicine, University of Peradeniya, Peradeniya, Sri Lanka; c Pharmacology, Toxicology & Therapeutics, University/BHF Centre for Cardiovascular Science, University of Edinburgh, Edinburgh, UK; d Faculty of Medicine, University of Kelanyia, Kelanyia, Sri Lanka; e Department of Community Medicine, Faculty of Medicine & Allied Sciences, Rajarata University of Sri Lanka, Saliyapura, Anuradhapura, Sri Lanka; f Centre for Suicide Research, Department of Psychiatry, University of Oxford, Oxford, UK; g Department of Public Health, Faculty of Health and Medical Sciences, University of Copenhagen, Copenhagen, Denmark; h National Institute for Health Research Bristol Biomedical Research Centre, University Hospitals Bristol NHS Foundation Trust and University of Bristol, UK

**Keywords:** Suicide, Sri Lanka, Socioeconomic position, Self-harm, Asia, Multilevel models

## Abstract

**Background:**

An individual's suicide risk is determined by personal characteristics, but is also influenced by their environment. Previous studies indicate a role of contextual effects on suicidal behaviour, but there is a dearth of quantitative evidence from Asia.

**Methods:**

Individual and community level data were collected on 165,233 people from 47,919 households in 171 communities in rural Sri Lanka. Data were collected on individual (age, sex, past suicide attempts and individual socioeconomic position (SEP)) and household (household SEP, pesticide access, alcohol use and multigenerational households) level factors. We used 3-level logit models to investigate compositional (individual) and contextual (household/community) effects.

**Results:**

We found significant variation between households 21% (95% CI 18%, 24%) and communities 4% (95% CI 3%, 5%) in the risk of a suicide attempt. Contextual factors as measured by low household SEP (OR 2.37 95% CI 2.10, 2.67), low community SEP (OR 1.45 95% CI 1.21, 1.74), and community ‘problem’ alcohol use (OR 1.44 95% CI 1.19, 1.75) were associated with an increased risk of suicide attempt. Women living in households with alcohol misuse were at higher risk of attempted suicide. We observed a protective effect of living in multigenerational households (OR 0.53 95% CI 0.42, 0.65).

**Limitations:**

The outcome was respondent-reported and refers to lifetime reports of attempted suicide, therefore this study might be affected by socially desirable responding.

**Conclusions:**

Our study finds that contextual factors are associated with an individual's risk of attempted suicide in Sri Lanka, independent of an individual's personal characteristics.

## Highlights

• A person's environment influences their suicide risk.
• There is a dearth of quantitative studies from Asia investigating contextual factors.
• Suicide risk in rural Sri Lanka is associated with low community and household SEP.
• Community ‘problem’ alcohol use increases the risk of lifetime suicide attempts.
• Women in households with alcohol misuse are at a higher risk of attempted suicide.

## 1 Introduction

In the early 19th century, even before the seminal work by Durkheim(1951), there was a recognition that an individual's suicide risk is not only determined by individual characteristics, but also influenced by the society within which they live (Goldney et al., 2008). Area factors associated with suicidal behaviour include social fragmentation, socioeconomic deprivation, and unemployment (Milner et al., 2013, Rehkopf and Buka, 2006). Smaller scale qualitative studies have also shown the importance of a person's environment in contributing to their risk of suicidal behaviour, but these studies are limited in their generalisability (Dongre and Deshmukh, 2012, Widger, 2015b). The limitation of many of the previous quantitative studies (including Durkheim's work) is that the data were either collected at an area or an individual level, and the two levels of data were not combined. These studies have been criticised for not being able to disentangle whether the area effect observed is due to the characteristics of the individuals living in these areas (compositional^1^), or because of characteristics of the area itself (contextual^2^), over and above that due to its composition.

Statistical techniques are now available that can distinguish compositional or contextual effects allowing us to investigate whether individuals living in areas of concentrated poverty have a higher risk of suicide regardless of their own level of poverty. Multilevel modelling techniques allows the researcher to model individual relationships, group relationship, and the link between them. Studies have investigated the influence of compositional and contextual effects simultaneously on suicide risk in high income countries (HIC) (Agerbo et al., 2007, Collings et al., 2009, Denney et al., 2015, Hawton et al., 2001, Johnston et al., 2006, Maimon and Kuhl, 2008, Martikainen et al., 2004, Neeleman and Wessely, 1999, O'Reilly et al., 2008, Turnbull, 2014, Zammit et al., 2014), with few studies investigating attempted suicide risk (Hawton et al., 2001, Maimon and Kuhl, 2008). There is evidence from these studies that contextual factors influence suicide risk (Agerbo et al., 2007, Denney et al., 2015, Hawton et al., 2001, Martikainen et al., 2004, Zammit et al., 2014). To the best of our knowledge there have been no similar investigations in low and middle income countries (LMIC). The present study investigates the effect of contextual factors on suicide attempt risk in a LMIC. The influence of contextual factors may be more pronounced in these settings because of the heavy emphasis on family relationships and collectivism (Bolz, 2002). In collectivist cultures the family unit and its characteristics form a strong part of a person's identity and how they are viewed by other society members. Konradsen et al. describe cases where young men attempted suicide as a consequence of their father's shameful behaviour becoming known in the community (Konradsen et al., 2006). The investigation of the role of families and community in suicide attempts is important, especially as family conflicts are one of the key triggers for attempts (Bolz, 2002, Konradsen et al., 2006, Marecek, 2006, Widger, 2015b).

Another important consideration when examining a person's environment is the degree to which that person's socioeconomic position (SEP) is compatible/comparable with the SEP status of their neighbours, and can lead to feelings of relative deprivation. Relative deprivation is when an individual judges their status in relation to others around them and decides that their status is at odds with their environment (Smith et al., 2012, Wilkinson, 1997). They may not be truly deprived in absolute terms. Differences between individual relative to area characteristics as a contributor to suicidal behaviour have been previously shown (i.e. cross-level interaction) (Neeleman and Wessely, 1999).

We investigated whether the risk of attempted suicide varied across household and/or community in rural Sri Lanka. We also investigated whether variations observed were due to compositional and/or contextual effects. This included looking at whether the context (household and community) within which a person lives was independently associated with attempted suicide risk, over and above that due to the composition of the household or community. Lastly, we investigated whether the effect of household/community SEP differs by an individual's own SEP (i.e. relative deprivation).

## 2 Methods

### 2.1 Context

Sri Lanka is an island situated off the south-east coast of India, with a population of 20 million (Census 2011). A large proportion of the population live in rural areas and engage in agriculture. The main ethnic group in Sri Lanka are the Sinhalese followed by Tamils. Historically, Sri Lanka had one of the highest suicide rates in the world (Knipe et al., 2017a), with higher rates observed in rural areas (Knipe et al., 2017b).

### 2.2 Participants

The data used in this study were collected as part of the baseline survey of a large community-based randomised controlled trial in the Anuradhapura district, North Central province of Sri Lanka (Safe Storage) (Pearson et al., 2017). This is primarily an agricultural area, with a Sinhalese majority. Data used for this analysis were collected between 31/12/2010 and 02/02/2013. All individuals living in the study area were eligible for inclusion. The details of the baseline survey have been described previously (Knipe et al., 2017c). Briefly, we conducted a door-to-door household interview survey. A face-to-face interview was performed with an adult (≥ 18 years) household member within their home compound in their local language (Sinhala or Tamil). After obtaining verbal consent, one (and sometimes more than one) member of the household was interviewed. Data collectors received regular refresher training, audits and feedback on data quality (Knipe et al., 2014b).

For trial logistic purposes the study area was split into 10 regions/bands. We only included data collected from bands 2–10, as the data in band 1 for our outcome (suicide attempt) and one of the SEP measures (household construction) were collected using slightly different definitions.

### 2.3 Data structure

**Fig. 1:**
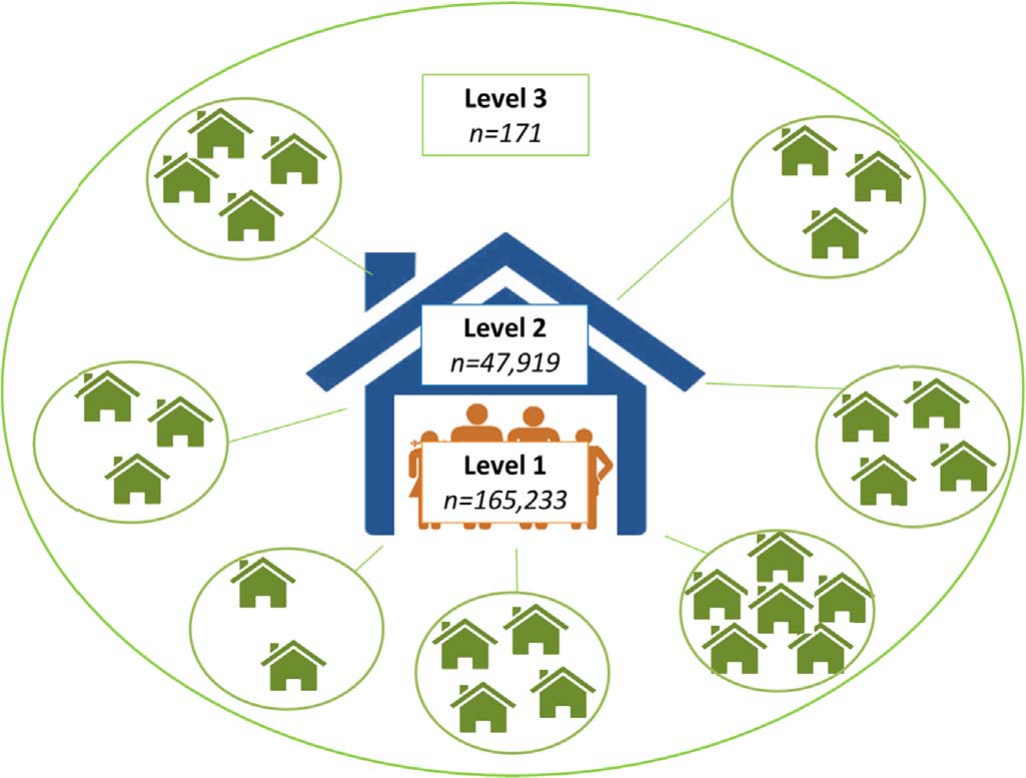
Data structure and number of units per level.

The data collected are clustered in nature: individuals (level 1, n = 165,233) within households (level 2, n = 47,919) within areas (level 3, n = 171) (Fig. 1). For this analysis we have used the cluster boundaries defined by the Safe Storage trial to define the level 3 units (i.e. communities) (Pearson et al., 2011). Briefly these boundaries were assigned based on geographic separation, membership of householders to particular organisations/temples, and local knowledge of village boundaries. Our on-the-ground knowledge from the fieldwork enabled us to define distinct geographical areas which related to real communities.

### 2.4 Data collected

The survey included data on characteristics of the household and each household member. We also recorded data on lifetime suicide attempts reported by household members. Survey data were recorded directly onto handheld devices (Knipe et al., 2014b). Using the data collected we defined contextual (both derived and measured at levels 2 and 3) and compositional (level 1) variables.

#### 2.4.1 Individual measures – level 1

##### 2.4.1.1 Age and sex

We collected data on sex and age or date of birth where available for each household member. Age was categorised into 5 groups: 0–9; 10–25; 26–40; 41–55; and 56+ years of age. These age groups were based on the age-specific incidence of suicide attempts within the dataset.

##### 2.4.1.2 Individual SEP (education)

For each household member the respondent was asked to report the completed level of education and qualification received. For younger participants the current education was recorded. This was categorised into five groups: i) not attended school; ii) primary education only; iii) completed ordinary level (O-level) - examinations are taken around the age of 16 years; iv) completed advanced level (A-level) – A-level examinations taken around the age of 18 years; and v) university. There were relatively few individuals with a university education (n = 2740, 1.7%) and we combined this category with those with an A-level qualification (n = 27410, 16.6%).

##### 2.4.1.3 Lifetime suicide attempts

Survey respondents were asked the following question: *“Has anyone in this household attempted suicide?”*. All suicide attempts were recorded, regardless of method. The household member(s) who attempted suicide were recorded when possible, enabling us to identify the individual household member who attempted suicide in 88% of cases. Only non-fatal attempts were included in this analysis. Most life-time suicide attempts were due to pesticide self-poisoning (62%).

#### 2.4.2 Household measures – level 2

##### 2.4.2.1 Household SEP

A composite asset score was derived by combining data on household construction and motorised vehicle ownership. Households were categorised by data collectors into three according to the principal materials used in the construction of walls, roof and floor: i) durable (e.g. bricks, cement); ii) non-durable (e.g. mud, straw); or iii) a combination of durable and non-durable materials. Households were asked whether or not they had a 2–3 wheel (e.g. motorbike or three-wheeler) vehicle, and/or a 4 wheel vehicle (e.g. car or tractor). We created a single asset score by dichotomising motorised vehicle ownership and household construction. These categories allowed us to create a variable with three levels – low (no motorised vehicle AND poor quality household construction); middle (either a motorised vehicle OR moderate/high quality household construction); or high (motorised vehicle ownership AND moderate/high quality household construction) asset ownership.

##### 2.4.2.2 Household pesticide access

We collected data on pesticide storage and use. We used this to generate a binary variable to indicate whether a household had access to pesticides. Access was defined as either storing pesticide within the home compound (home garden, separate shed or within the home) or using pesticides (home garden, or seasonal use) or having no use/access. We included pesticide exposure in this analysis because previous studies have found an association of environmental pesticide exposure with an increased likelihood of suicidal ideation (Zhang et al., 2009).

##### 2.4.2.3 Absence of young children in the household

Evidence from high income countries suggest that parents are at a decreased risk of suicide, especially if the children are young (Qin and Mortensen, 2003). We identified households with young children (9 years and younger).

##### 2.4.2.4 Number of generations in a household

Multigenerational households in Asia are common. However, in modern day Sri Lanka the existence of multigenerational households may increasingly lead to difficult family environments. Sri Lankan society is built on strong cultural norms, generational and gender hierarchies. Young people in Sri Lanka are being increasingly exposed to more western ideals through social media such as Facebook and the internet. This has led to a shift in expectations. This shift in expectations can lead to arguments with family members (Marecek and Senadheera, 2012). Based on the hierarchies that exist in a household, children should not confront, disagree or show strong emotions in front of their elders (Marecek, 2006). The resulting retaliation can take the form of attempted suicide, which is a culturally acceptable form of communicating distress (Chapin, 2014, Widger, 2015a). As we did not collect information on household family relationships, we created an indirect measure of the number of generations in a household by splitting the occupants into four age categories: < 10; 10–25, 26–65 and 65+. We then counted how many of these age categories there were in each household: 1, 2, 3 or 4. We acknowledge that this is a simplification as it is highly likely that for families with several children there will be children in both the first two age categories.

##### 2.4.2.5 ‘Problem’ alcohol use

The household respondent(s) were asked whether someone in the household consumed alcohol, and then whether this alcohol consumption was perceived by anyone in the household as a ‘problem’. This was asked in a single question and no prompts were given as to what would be considered as problem use. The survey did not ask about individual level alcohol consumption or misuse. This measure does not relate to a medical definition of alcoholism.

#### 2.4.3 Community measures – level 3

##### 2.4.3.1 Area level deprivation

Area level deprivation was derived by aggregating a measure of household SEP (asset score) up to the community level. The percentage of households with a poor asset score was derived for each community and categorised into quintiles.

##### 2.4.3.2 Pesticide exposure

Area level pesticide exposure was derived by aggregating household pesticide exposure/access (household pesticide access) up to the community level. The percentage of households with access to pesticides (as a proxy measure to environmental pesticide exposure) was derived for each community, and categorised into quintiles.

##### 2.4.3.3 Community alcohol misuse

Community level alcohol misuse was derived by aggregating the measure of alcohol problem in the household up to the community level. The percentage of households with a reported alcohol problem was derived for each community, and categorised into quintiles. Community level alcohol misuse may increase suicidal behaviour because of social fragmentation or increased community level violence (Evans et al., 2004).

### 2.5 Statistical analysis

Modelling household and community level variables (level 2 & 3) simultaneously with a similar factor(s) at a lower level(s) (e.g. community deprivation, household and individual SEP) allows us to pick out potential contextual community/household effects over and above the effect of the same variable acting at a household/individual (i.e. due to the composition) level. Not all household measures have a complimentary individual level variable (e.g. alcohol misuse/ pesticide access).

In this analysis we use multilevel models which attempt to distinguish variation in outcome at the different levels, i.e. individual (by convention referred to as level 1), household (level 2) and community (level 3). Given the outcome of interest is binary, the data were analysed using multilevel mixed effects logistic regression using the *melogit* Stata command. The multilevel modelling command in Stata 14 uses a direct maximum likelihood estimation (we employed the 12 quadrature point option).

We first fitted a variance component model (i.e. a multilevel model with no explanatory variables) in order to estimate the variance partition coefficient (VPC). This is an estimate of the proportion of variation attributed to each level of the data structure, and has been previously been used in suicide research (Zammit et al., 2014). This is sometimes interpreted as the “importance” of each level (i.e. the higher the accounted variance, the more “important” that level is) (Goldstein et al., 2002). In order to calculate a VPC for a logistic multilevel model, it is common practice to use the latent response formulation (Goldstein et al., 2002). We did not calculate a VPC for the lower level unit (individuals) as this is fixed in the latent response formulation. This means that we are not able to directly estimate the VPC for level 1 nor are we able to compare the VPC between models because any observed changes may not be due to real changes but to latent variable scale changes (Hox, 2010).

We fitted a model with all the individual level factors and then added in each household level variable in a stepwise fashion. We only retained factors which improved the fit of the model for these data (identified through likelihood ratio tests- threshold of p < .05). The final model included all individual variables, household variables which improve fit to the data, and all community level factors. We also fitted an additional model adjusting for respondent type (head of household or other household member). As this is a cross-sectional analysis we have estimated odds ratios, but given that the risk of attempted suicide is small (less than 0.1), the odds are very close to the risk and therefore we have interpreted the odds ratios as risk ratios (Kirkwood and Sterne, 2003).

Due to differences in the risk of attempted suicide between males and females, we examined whether there was evidence of differences between males and females in the associations between each risk factor and attempted suicide by adding interaction terms to the models.

We also investigated the concept of relative deprivation, by fitting two cross-level interaction models. The two models incorporating interaction terms allow for the effect of household SEP (asset score) and community SEP (deprivation) on an individual's risk of attempted suicide to depend on an individual's own SEP (education). We did this by fitting a model with several interaction terms between individual SEP and household SEP, and then did a likelihood ratio test to determine whether the inclusion of these cross level interaction terms improved the model fit to these data. We did the same procedure to test for an interaction between individual SEP and community SEP.

Given the rarity of attempted suicide, we chose to use lifetime suicide attempts as the outcome of interest in order to limit the degree of violation of model assumptions (i.e. normal distribution of household level residuals). As discussed later (see discussion) mis-specifying the model can lead to an overestimation of household level variance but will have limited impact on the fixed parameter estimates (i.e. effect estimates). As the SEP measures at baseline are more likely to be related to the respondent-reported suicide attempts in the last year, we fitted a multilevel model to see whether a similar SEP association to that of lifetime attempts was observed. Any adult household member was able to respond to the survey and therefore it is possible that respondents may differentially report suicide attempts or problem alcohol use. We therefore investigated the impact of respondent type on the associations observed.

### 2.6 Ethics

Ethics approval was granted from the University of Peradeniya and the ethical review committee of the Faculty of Medicine and Allied Sciences, Rajarata University Sri Lanka.

## 3 Results

The median number of households in each community was 259 (interquartile interval (IQI): 202, 322) and the number of individuals was 918 (IQI 692, 1144). The median number of individuals in a household was 3 (IQI 2, 4). Table 1 presents the basic description of the sample.

**Table 1:** Descriptive characteristics of the sample.

	n (%)		
	Individuals	Households	Communities
N =	165233	47919	171
*Individual factors*			
Male gender	81252 (49.2)		
Age group			
10–25	50533 (30.6)		
26–40	52171 (31.6)		
41–55	36968 (22.4)		
55+	25561 (15.5)		
Education			
University/A-level	30150 (18.3)		
O-level	105403 (63.8)		
Primary	24542 (14.9)		
Not attended	5138 (3.1)		
*Household factors*			
Asset score			
High	107324 (65.0)	29387 (61.3)	
Moderate	48368 (29.3)	15154 (31.6)	
Low	9541 (5.8)	3378 (7.1)	
No. of generations in the household			
1	13019 (7.9)	5901 (12.3)	
2	80514 (48.7)	24082 (50.3)	
3	61957 (37.5)	15971 (33.3)	
4	9743 (5.9)	1965 (4.1)	
Household alcohol 'problem'	44616 (27.0)	12318 (25.7)	
Household pesticide access	133101 (80.6)	37630 (78.5)	
Household without children	80213 (48.6)	22807 (47.6)	
*Area factors*			
Deprivation^a^			
0–8%	32454 (19.6)	9302 (19.4)	39 (22.8)
9–10%	33350 (20.2)	9604 (20)	27 (15.8)
11–14%	33253 (20.1)	9560 (20)	37 (21.6)
15–18%	32920 (19.9)	9588 (20)	30 (17.5)
19–56%	33256 (20.1)	9865 (20.6)	38 (22.2)
Alcohol "problem"^b^			
1–20%	32040 (19.4)	9233 (19.3)	35 (20.5)
21–23%	33321 (20.2)	9663 (20.2)	33 (19.3)
24–26%	33050 (20.0)	9589 (20.0)	36 (21.1)
27–30%	33097 (20.0)	9560 (20.0)	37 (21.6)
31–46%	33725 (20.4)	9874 (20.6)	30 (17.5)
Pesticide access^c^			
19–73%	32784 (19.8)	9506 (19.8)	34 (19.9)
74–77%	32003 (19.4)	9360 (19.5)	27 (15.8)
78–83%	33794 (20.5)	9746 (20.3)	35 (20.5)
84–87%	33473 (20.3)	9717 (20.3)	35 (20.5)
88–98%	33179 (20.1)	9590 (20.0)	40 (23.4)

a % of households with a low asset score categorised into quintiles.

b % of households with 'problem' alcohol use categorised into quintiles.

c % of households with access to pesticides categorised into quintiles.

There were 3681 (1810 male; 1871 female) individuals with a respondent-reported lifetime suicide attempt. This gave a risk of 22.3 per 1000 in ≥ 10 year olds; a similar risk was observed in males and females. Young people (current age 26–40 year olds) had the highest risk of a lifetime suicide attempt (30 per 1000). Roughly 7% (n = 3463) of households included at least one person who had attempted suicide, with only 0.5% (n = 218) of households reporting more than 1 person attempting. The majority of communities (99% n = 169) had more than 1 attempt. There were no communities with no lifetime suicide attempts. Based on the VPC, 4% (95% CI 3%, 5%) of the total variance in the prevelance of lifetime suicide attempts, was accounted for by variation at the community level and 21% (95% CI 18%, 24%) at the household level.

Using a stepwise approach the following household factors were retained in the model: 1) household SEP (asset score); 2) number of generations in a household; and 3) household alcohol problem. The likelihood ratio test indicated that the inclusion of absence of children (p = .56) and household pesticide access (p = .23) did not improve the fit of the model to the data. The final model controls for compositional and contextual factors simultaneously, and provides evidence that both household and community SEP are associated with an individual's lifetime suicide attempt independent of individual SEP (Table 2). In other words, there was evidence that living in a household with poorer assets (household context) was associated with a lifetime suicide attempt (OR 2.37), over and above that due to the SEP of the individual as indexed by their education (Table 2). Individuals with low SEP were, however, still at increased risk of lifetime attempted suicide. The analysis of the role of household composition in relation to the number of generations living together indicates that living in a household with more generations reduces the associated risk of an individual attempting suicide by as much as 47% (OR 0.53 95% CI 0.42, 0.65).

**Table 2:** Association of compositional, contextual factors and lifetime attempted suicide risk, overall and by sex (models are adjusted for all factors presented).

	OR (95% CI)			P-value for interaction*
	All	Female	Male	
*Individual factors*				
Male gender	0.95 (0.89,1.01)			
Age group				
10–25	1	1	1	
26–40	1.84 (1.69,2.01)	1.40 (1.25,1.57)	2.62 (2.28,3.01)	
41–55	1.20 (1.08,1.33)	0.76 (0.66,0.88)	1.98 (1.70,2.31)	
55+	0.53 (0.46,0.61)	0.25 (0.20,0.32)	1.09 (0.90,1.33)	
Education				
University/A-level	1	1	1	
O-level	2.10 (1.85,2.37)	1.94 (1.66,2.26)	2.75 (2.21,3.43)	
Primary	3.27 (2.82,3.80)	2.67 (2.19,3.27)	4.72 (3.71,6.01)	<0.001
Not attended	3.22 (2.60,3.98)	2.99 (2.25,3.96)	5.20 (3.72,7.28)	
*Household factors*				
Asset score				
High	1	1	1	
Moderate	1.61 (1.50,1.74)	1.50 (1.36,1.67)	1.70 (1.53,1.90)	0.004
Low	2.37 (2.10,2.67)	2.07 (1.75,2.45)	2.57 (2.19,3.03)	
No. of generations in the household				
1	1	1	1	
2	0.79 (0.69,0.89)	0.78 (0.65,0.93)	0.79 (0.67,0.93)	
3	0.79 (0.70,0.90)	0.76 (0.63,0.92)	0.78 (0.66,0.93)	0.933
4	0.53 (0.42,0.65)	0.53 (0.39,0.71)	0.52 (0.38,0.70)	
Household "alcohol" problem	2.31 (2.15,2.48)	1.96 (1.77,2.16)	2.66 (2.41,2.95)	<0.001
*Area factors*				
Deprivation**				
0–8%	1	1	1	
9–10%	1.27 (1.05,1.55)	1.24 (0.99,1.55)	1.33 (1.04,1.70)	
11–14%	1.02 (0.84,1.23)	1.00 (0.80,1.24)	1.05 (0.82,1.33)	0.484
15–18%	1.23 (1.02,1.49)	1.18 (0.95,1.47)	1.30 (1.02,1.65)	
19–56%	1.45 (1.21,1.74)	1.36 (1.10,1.68)	1.52 (1.20,1.92)	
Alcohol "problem"***				
1–20%	1	1	1	
21–23%	1.18 (0.98,1.43)	1.16 (0.93,1.44)	1.19 (0.93,1.51)	
24–26%	1.09 (0.90,1.32)	1.08 (0.86,1.34)	1.08 (0.85,1.37)	0.736
27–30%	1.14 (0.95,1.38)	1.08 (0.87,1.34)	1.21 (0.95,1.53)	
31–46%	1.44 (1.19,1.75)	1.46 (1.18,1.81)	1.40 (1.10,1.78)	
Pesticide access****				
19–73%	1	1	1	
74–77%	0.94 (0.77,1.14)	0.81 (0.65,1.01)	1.10 (0.86,1.41)	
78–83%	0.95 (0.79,1.14)	0.80 (0.65,0.99)	1.12 (0.89,1.41)	0.001
84–87%	0.93 (0.77,1.12)	0.85 (0.69,1.05)	1.01 (0.79,1.28)	
88–98%	1.01 (0.84,1.21)	0.91 (0.74,1.12)	1.13 (0.90,1.42)	

* Likelihood ratio test to indicate whether the inclusion of an interaction term with sex improved data fit.

** % of households with a low asset score categorised into quintiles.

*** % of households with 'problem' alcohol use categorised into quintiles.

**** % of households with access to pesticides categorised into quintiles.

Living in areas with higher levels of deprivation was associated with a higher risk of a lifetime attempted suicide (OR in highest quintile 1.45 95% CI 1.21, 1.74), whereas there was no evidence that living in areas with higher levels of pesticide access increased risk (Table 2). There was evidence that living in a community with a high percentage of households with a self-reported alcohol problem increased the risk of a lifetime suicide attempt by 44% (OR in highest quintile, 1.44 95% CI 1.19, 1.75), independently of whether the individual was from a household with a reported alcohol problem (Table 2).

Table 2 presents the association of each factor included in the final model stratified by sex. There is statistical evidence that some of the associations are modified by sex. The most striking differences between men and women are with the associations between education and risk of lifetime attempted suicide (1.4–1.8 fold differences), with a stronger association seen in men than women.

Table 3 presents the association of individual level SEP stratified first by household SEP and then community level deprivation (community SEP). There was some evidence that individuals living in households with a higher household SEP than their own SEP status had an increased odds of a lifetime attempt, the greatest risk (OR 4.33) was seen amongst those who had not attended school but lived in a high SEP household. However, that subgroup was very small, and the formal test did not provide statistical evidence for either cross-level interaction (Table 3).

**Table 3:** Cross-level interactions of SEP with a lifetime suicide attempt.

	Education (Individual SEP) OR (95% CI)				P value for interaction
	University /A-level	O-level	Primary	Not attended	
Asset score (Household SEP)					
High	1	2.08 (1.79, 2.43)	3.36 (2.76, 4.10)	4.33 (3.15, 5.94)	0.12*
Moderate	1	1.95 (1.55, 2.47)	3.04 (2.34, 3.94)	2.89 (2.04, 4.09)	
Low	1	1.72 (0.92, 3.21)	2.52 (1.32, 4.82)	1.99 (0.97, 4.10)	
Area deprivation in quintiles					
(Area SEP)***					
0–8%	1	2.40 (1.81, 3.19)	3.29 (2.31, 4.69)	4.05 (2.41, 6.79)	0.55**
9–10%	1	1.77 (1.37, 2.29)	2.69 (1.96, 3.68)	3.12 (1.94, 5.03)	
11–14%	1	2.07 (1.54, 2.77)	3.06 (2.14, 4.37)	2.5 (1.41, 4.44)	
15–18%	1	2.18 (1.63, 2.91)	4.09 (2.93, 5.71)	3.54 (2.18, 5.74)	
19–56%	1	1.95 (1.48, 2.57)	3.04 (2.23, 4.16)	3.02 (2.03, 4.49)	

Likelihood ratio test between a model with and without interaction parameters for:.

* Education and asset ownership (cross level interaction between individual and household level).

** Education and area level deprivation (cross level interaction between individual and area level).

*** % of households with a low asset score categorised into quintiles.

Adjusting for type of household respondent (head of household vs. other household member; and the sex of the respondent) did not alter the results (data not shown). We repeated the analysis on suicide attempts occurring in the last year and found similar associations (Supplementary Table 1).

## 4 Discussion

This is the first study to investigate the effect of community and household characteristics on respondent reported lifetime risk of attempted suicide in a general rural population sample in a LMIC. We found that there was significant variation between households and communities in the odds of a lifetime suicide attempt; 25% of the total variance was estimated to be due to higher level units (i.e. households and communities). The findings suggest that contextual factors such as household/community SEP and community level problematic alcohol use are associated with an increased risk of a suicide attempt, over and above that which would be expected due to the individuals living in these households or communities. In addition we found that individuals living in households with several generations were less likely to have reported an attempted suicide. The cross-level interaction models found no convincing evidence to support the theory of relative deprivation being an important risk factor for suicide attempt.

### 4.1 Strengths and limitations

This study is based on a large representative dataset from a rural community in a LMIC. The study achieved a very high response rate (95%) and included data on a range of SEP indicators. The results, however, should be interpreted in light of several limitations. First, the outcome was respondent-reported and refers to lifetime reports of attempted suicide. It is possible that some of the associations we observe may be a consequence of socially desirable responding. Individuals from higher SEP backgrounds may be less likely to report a suicide attempt than individuals from a lower SEP status. In addition, because we asked about lifetime suicide attempts, it is possible that the respondent would not know about attempts made by another household member many years ago. This could be socially patterned especially because households from a lower SEP background were more likely to have the head of household responding. We tested for this by adjusting for the type of respondent (head of household vs. non-head of household) but the adjusted analysis showed no evidence that respondent type impacted on the associations we observed. In addition, we were only able to investigate the association with suicide attempts and not deaths because demographic details of individuals who died before the baseline survey were not collected. Due to time constraints we were unable to give households a detailed definition of what would be considered a suicide attempt nor were we able to assess suicidal intent. It may be that we have included non-suicidal self-injury, which is potentially a different behaviour, within our category of those who attempted suicide.

Second, problem alcohol use was recorded from a single question and not a validated questionnaire, and this was only recorded at a household level. We are, therefore, unable to pick out whether the association of problem household alcohol consumption created a unique environmental context in the household which increased the risk of a suicide attempt, or whether the association observed was due to those individuals in the household with an alcohol problem being at higher risk of attempted suicide. The sex stratified analysis, however, suggests that at least for women, problem alcohol use at the household level is associated with an increased risk of attempted suicide, as very few Sri Lankan women drink alcohol (Sørensen et al., 2014).

Third, the associations observed may be due to reverse causality as the data are cross-sectional. In other words the information on attempted suicide is based on lifetime suicide attempts the individual and contextual factors might have changed over time and the SEP at the time of the survey may not reflect the SEP status at the time of the attempt. The suicide attempt of a household member may lead to a reduction in the household's SEP because of loss of income, it may also lead to a social drift of these households into areas of concentrated poverty. We ran the full model with suicide attempts in the last year as the outcome measure and found similar associations to our main analysis.

Fourth, we are unable to conclude exactly what it is about living in areas of higher levels of deprivation and problem alcohol use that gives rise to the higher level of attempted suicide in these areas. Qualitative investigation is needed to fully understand what these variables mean in order to help inform suicide prevention efforts. In addition, the contextual factors (especially at the community level) were derived from individual/household level factor and are not truly contextual in the way that infrastructure indicators are (e.g. number of school or health clinics).

Lastly, the rarity of the outcome means that the residuals at the household level variance are non-normally distributed, which violates the assumption of a multilevel logistic regression model. The consequence is that we are likely to have overestimated the variation (21%) at the household level (Agresti et al., 2004). There are currently no suitable non-parametric multivariate methods available to model this type of outcome (Agresti et al., 2004). The degree of overestimation is unknown but is unlikely to be sizeable. One UK study investigating contextual risk factors for common mental disorders (a risk factor for suicide) estimated that 19% (95% CI 14–24%) of the total variation of common mental disorders was attributed to the household level (Weich et al., 2003); this is consistent with the amount of variation attributed to the household (21% 95% CI 18–24%) for lifetime suicide attempts in our study. Given that common mental disorders are detected more frequently, the normal assumption regarding the household residuals is unlikely to be violated. Whilst the misspecification of the model may have led to an overestimation of the random part of the model (i.e. the household variance), the fixed parameter estimates (i.e. odds ratios) if they are not too large (i.e. OR > 7) will be reasonably well estimated (Agresti et al., 2004).

### 4.2 Comparison to other studies

The estimated variance of attempted suicide attributed to the community level factor (4% 95% CI 3–5%) in this study are consistent (though somewhat higher) with the two previous studies of suicide in HIC, which estimated this variance to be 0.76% 95% CI 0.05–3.99% (Zammit et al., 2014); and 3% (Denney et al., 2015). This support our hypothesis that contextual factors may be more important in LMIC. Previous studies have shown that contextual effects, such as socioeconomic deprivation and low social/family cohesion, increase the risk of suicide mortality independent of individual factors (Collings et al., 2009, Denney et al., 2015, Martikainen et al., 2004). This is consistent with the findings of our analysis which found that higher levels of deprivation, lower levels of social support (fewer cohabiting generations), and higher levels of problem alcohol consumption (possibly a proxy for social fragmentation) were independently associated with a higher odds of attempted suicide. Several previous studies did not show an independent association of contextual factors once compositional factors were included in analytical models (Maimon and Kuhl, 2008, Neeleman and Wessely, 1999, O'Reilly et al., 2008, Zammit et al., 2014). Possible explanations could be: 1) that the difference reflects cultural differences; 2) methodological differences due to the size of the area unit used (i.e. large areas vs. smaller neighbourhoods); 3) the inclusion of several factors in the model which are highly correlated (at the same level) and which therefore attenuate the associations towards the null. Few studies have investigated whether the contextual socioeconomic environment modified the association of individual SEP on suicide risk (Agerbo et al., 2007, Martikainen et al., 2004, Zammit et al., 2014). Consistent with this analysis, they showed no strong statistical evidence of a cross-level interaction. Our findings, however, are contrary to finding from previous studies in Sri Lanka exploring the area level variation of suicide (Knipe et al., 2017b) and self-poisoning (Hanwella et al., 2013, Manuel et al., 2008). These ecological studies find areas with increased deprivation had a lower rate of suicide or self-poisoning. One possible explanation for our opposing results is that we were able to simultaneously model the contribution of compositional factors (i.e. individual) with community (i.e. area) factors. Another possible explanation is that the epidemiology of suicidal behaviour has dramatically changed over the last few decades (Knipe et al., 2014a).

In Sri Lanka, like many South Asian countries, the prevalence of alcohol use by females is extremely low (Sørensen et al., 2014). In light of this, for women the problem alcohol use of the household represents a risk associated with the household context and not the composition, as women themselves are unlikely to be consuming alcohol. The risk of attempted suicide is nearly double in women living in households with a household member who misuses alcohol after controlling for socio-economic variables. This is consistent with previous work from India which has indicated that increased levels of hazardous drinking in male household members increases the risk suicidal behaviour in co-habiting women (Gupta et al., 2015), and research from Sri Lanka indicating husband/father alcohol misuse as a preceding factor for suicidal behaviour (Gamburd, 2008, Konradsen et al., 2006, Marecek, 2006; Sørensen et al., 2017) Problematic alcohol use is often associated with intimate partner violence in Sri Lanka (Abeyasinghe, 2002, Gamburd, 2008). It may be that the increased risk of attempted suicide seen in women in households with problem alcohol use is due to these women experiencing such violence. Possible targets for suicide prevention efforts in this setting could be in reducing the prevalence of problematic alcohol consumption and intimate partner violence.

Contrary to our original hypothesis that living in a multigenerational household would increase the risk of attempted suicide, especially in young people, we find the opposite effect - living in households with more generations reduces the risk. This reduction in risk might reflect the fact that individuals in these households have increased levels of social support and/or be because suicide attempts are more likely to be halted because of older family members being present in the household when the suicide attempts takes place.

We found no evidence in our analysis that access to pesticides in the home or increased pesticide exposure in communities increased the risk of a lifetime attempt of suicide. This is inconsistent with previous evidence which indicated that household pesticide storage increased the odds of suicidal ideation (Zhang et al., 2009). The inconsistency may be because in our study the majority of individuals lived in households with access to pesticides (81%) and therefore it is possible that we do not have enough unexposed individuals to be able to detect a difference. It could also be that an association between pesticide access and suicide attempts is partly obscured by the high case-fatality of this method during the period prior to the baseline survey.

## 5 Conclusion

Multilevel modelling of the risk of attempted suicide indicated that household and community level factors (i.e. contextual factors) played an important role in influencing an individual's risk of an attempted suicide, independent of an individual's personal characteristics. We found that nearly a quarter of the variation in this Sri Lankan dataset is attributed to the household and community level. Higher rates of attempted suicide were seen in more deprived household and community environments. However, individuals in multigenerational households had a reduced risk of attempted suicide. There was also evidence that communities with higher levels of problem alcohol use increased the associated risk of attempted suicide. This study highlights possible areas for community intervention, but we are unable to conclude what these intervention strategies should be. A further qualitative investigation of the meaning of observed contextual associations with attempted suicide risk will be needed.

## Footnotes

1 Compositional factors are measures of the individual (e.g. education) and are later referred to as level 1 factors.

2 Contextual factors are variables that relate to the environment and later referred to as level 2 and 3 factors. Ideally these are measured at the relevant level (i.e. area for level 3 variables). An alternative approach to generate contextual factors is to aggregate lower levels characteristics up to a higher level, as done in this study.
