# Supplementary material for: Attempted suicide in Sri Lanka – An epidemiological study of household and community factors
Source: J Affect Disord. 2018 May;232:177–84. doi: 10.1016/j.jad.2018.01.028 (PMC6081369; doi:10.1016/j.jad.2018.01.028)
Supplement: Supplementary file 1 — Supplementary material [file mmc1.docx]

Supplementary table 1 - Association of compositional and contextual factors with suicide attempt risk in the last year

|  |  |  |  | OR (95% CI) |
| --- | --- | --- | --- | --- |
|  | Individual factors | | |  |
|  |  | Male gender | | 0.74 (0.61,0.91) |
|  |  | Age group | |  |
|  |  |  | 10-25 |  |
|  |  |  | 26-40 | 0.63 (0.51,0.79) |
|  |  |  | 41-55 | 0.27 (0.19,0.37) |
|  |  |  | 55+ | 0.07 (0.04,0.14) |
|  |  | Education | |  |
|  |  |  | University/A-level | 1 |
|  |  |  | O-level | 1.60 (1.17,2.18) |
|  |  |  | Primary | 2.19 (1.43,3.34) |
|  |  |  | Not attended | 3.11 (1.65,5.86) |
|  | Household factors | | |  |
|  |  | Asset score | | 1 |
|  |  |  | High | 1.65 (1.32,2.06) |
|  |  |  | Moderate | 2.52 (1.82,3.51) |
|  |  |  | Low |  |
|  |  | No. of generations in the household | |  |
|  |  |  | 1 | 1 |
|  |  |  | 2 | 0.58 (0.40,0.84) |
|  |  |  | 3 | 0.45 (0.31,0.67) |
|  |  |  | 4 | 0.38 (0.20,0.71) |
|  |  | Household "alcohol" problem | | 1.86 (1.51,2.28) |
|  | Area factors | | |  |
|  |  | Deprivation* | |  |
|  |  |  | 0-8% | 1 |
|  |  |  | 9-10% | 1.25 (0.86,1.81) |
|  |  |  | 11-14% | 0.96 (0.66,1.41) |
|  |  |  | 15-18% | 0.97 (0.66,1.42) |
|  |  |  | 19-56% | 1.38 (0.96,1.97) |
|  |  | Alcohol "problem" ** | |  |
|  |  |  | 1-20% | 1 |
|  |  |  | 21-23% | 1.09 (0.77,1.56) |
|  |  |  | 24-26% | 0.87 (0.60,1.27) |
|  |  |  | 27-30% | 1.06 (0.75,1.52) |
|  |  |  | 31-46% | 0.96 (0.67,1.37) |
|  |  | Pesticide access *** | |  |
|  |  |  | 19-73% | 1 |
|  |  |  | 74-77% | 0.91 (0.63,1.31) |
|  |  |  | 78-83% | 1.02 (0.72,1.44) |
|  |  |  | 84-87% | 0.90 (0.63,1.30) |
|  |  |  | 88-98% | 1.00 (0.70,1.42) |

* % of households with a low asset score categorised into quintiles; ** % of households with “problem” alcohol use categorised into quintiles; *** % of households with access to pesticides categorised into quintiles
